# To trust? Or to verify?

**DOI:** 10.3205/zma001198

**Published:** 2018-11-15

**Authors:** Sigrid Harendza

**Affiliations:** 1 Universitätsklinikum Hamburg-Eppendorf, III. Medizinische Klinik, Hamburg, Deutschland

## Editorial

One of the most important medical facets of competence is to take responsibility. Supervisors of newly graduated physicians would like to rely on their accountable actions from day one on the job [1]. It is postulated likewise that continuous integration of physicians in an interprofessional team during an early phase of training is essential to foster and strengthen their trust in the assumption of responsibility by others [2]. Relying upon all members of the health care system to fulfil their tasks by their own responsibility and according to current knowledge to the well of the patients is the basis of cooperation in hospitals, private practices and other medical institutions. That young physicians feel heavily burdened by taking responsibility and dealing with uncertainty when starting their work despite previous curricular reforms is as well-known as the fact, that the stress of transition into professional medicinal life is reduced by the amount of clinical experience gained during undergraduate medical training [3]. How can the current developments in design of undergraduate medical education be classified from these points of view? 

With the so-called initiative Masterplan for Medial Education 2020 (“Masterplan Medizinstudium 2020”) the German government intents together with the federal states to foster practical relevance during undergraduate medical training [Deutschlands Zukunft gestalten, Koalitionsvertrag zwischen CDU, CSU und SPD; https://www.cdu.de/sites/default/files/media/dokumente/koalitionsvertrag.pdf, retrieved 7.10.2018]. This provided an excellent instrument of control for curricular planners – when used in an optimal way – to foster assuming responsibility in medical students already during undergraduate education and ease their transition to postgraduate training. Different approaches are possible so that medical students learn to really take responsibility. The concept of “entrustable professional activities” (EPA), for instance, increasingly finds its way into the undergraduate medical curriculum and it is based on different levels of supervision [4]. On the other hand, the trust in a medical student to responsibly take over a certain task depends on the respective supervisor, the respective medical student, their relationship, the task itself and the context of the specific situation [5].

Many medical curricula are currently designed in modules and continuous supervision of individual students by the same teacher is hardly ever intended. Therefore, it will hardly ever be possible for many teachers to assess, whether a certain student can be entrusted with specific tasks. Furthermore, with an EPA-concept students benefit very much from continuous structured feedback, which is currently established in medical education only to a small extent. Additionally, undergraduate curricula in Germany provide rather summative than formative assessment formats. A stronger focus towards feedback- and competence-oriented education would require further steps towards faculty development with more courage towards subjectivity by the assessors at the same time [6]. Raising fears to lose the function of quality control by such ways of assessment are ill-founded insofar as especially formative assessments would open the possibility to react in a timely and differentiated way towards individual students’ competence deficits. One further aspect can be added: many teachers and assessors are little aware of their function as role models for medical students and the of importance that role models play for medical students as they progress through their undergraduate studies [7]. Therefore, the personal professional behavior plays an important role in medical education to be a credible teacher or assessor for the students. Thus, the path towards competence-based medical education requires great personal commitment by all participants.

Within this change of medical education and medical assessment, a number of steps have already been made, which are accompanied by research. In this edition of the GMS Journal for Medical Education Soemantri et al. demonstrate, for instance, which type of feedback is used in Mini Clinical Evaluation Exercises (Mini-CEX) and that faculty development is needed to use it correctly [8]. Dahmen et al. found that students saw an influence of Objective Structured Clinical Examinations (OSCE) on the learning and for the development of competences [9]. Ludwig and Ross discovered that, by the real experience of a general physician’s work in a rural area during the Practice Year, barriers against such work, which existed before the PY-trimester, could be reduced [10]. These examples of research demonstrate that in medical education, similar to postgraduate education [11], techniques of “parenthood” like role modelling behavior, learning through graduated responsibility and feedback will play a substantial role. The National Competence Based Catalogue of Learning Objectives for Undergraduate Medical Education (NKLM) [NKLM, http://www.nklm.de, accessed 2018-10-07], which currently undergoes further development, provides the optimal framework for this purpose [12].

## Competing interests

The author declares that she has no competing interests.
